# The Influence of Parental Health Literacy Status on Reach, Attendance, Retention, and Outcomes in a Family-Based Childhood Obesity Treatment Program, Virginia, 2013–2015

**DOI:** 10.5888/pcd14.160421

**Published:** 2017-09-28

**Authors:** Jamie M. Zoellner, Jennie Hill, Wen You, Donna Brock, Madlyn Frisard, Ramine Alexander, Fabiana Silva, Bryan Price, Ruby Marshall, Paul A. Estabrooks

**Affiliations:** 1 Department of Public Health Sciences, School of Medicine, University of Virginia, Charlottesville, Virginia; 2 Department of Epidemiology, University of Nebraska Medical Center, Omaha, Nebraska; 3 Department of Agricultural and Applied Economics, Virginia Tech, Blacksburg, Virginia; 4 Department of Human Nutrition, Foods, and Exercise, Virginia Tech, Blacksburg, Virginia; 5 Department of Nutrition, University of North Carolina at Chapel Hill, Chapel Hill, North Carolina; 6 Department of Health Promotion, Social and Behavioral Health, University of Nebraska Medical Center, Omaha, Nebraska; 7 University of Virginia Cancer Center, Charlottesville, Virginia; 8 Children’s Healthcare Center, Danville, Virginia

## Abstract

**Introduction:**

Few interventions have evaluated the influence of parent health literacy (HL) status on weight-related child outcomes. This study explores how parent HL affects the reach, attendance, and retention of and outcomes in a 3-month multicomponent family-based program to treat childhood obesity (iChoose).

**Methods:**

This pre–post, quasiexperimental trial occurred in the Dan River Region, a federally designated medically underserved area. iChoose research protocol and intervention strategies were designed using an HL universal precautions approach. We used validated measures, standardized data collection techniques, and generalized linear mixed-effect parametric models to determine the moderation effect of parent HL on outcomes.

**Results:**

No significant difference in HL scores were found between parents who enrolled their child in the study and those who did not. Of 94 enrolled parents, 34% were low HL, 49% had an annual household income of less than $25,000, and 39% had a high school education or less. Of 101 enrolled children, 60% were black, and the mean age was 9.8 (standard deviation, 1.3) years. Children of parents with both low and high HL attended and were retained at similar rates. Likewise, parent HL status did not significantly influence improvements in effectiveness outcomes (eg, child body mass index [BMI] *z* scores, parent BMI, diet and physical activity behaviors, quality of life), with the exception of child video game/computer screen time; low HL decreased and high HL increased screen time (coefficient = 0.52, standard error, 0.11, *P* < .001).

**Conclusion:**

By incorporating design features that attended to the HL needs of parents, children of parents with low HL engaged in and benefited from a family-based childhood obesity treatment program similar to children of parents with high HL.

## Introduction

Despite the public health priority for both preventing childhood obesity and increasing health literacy (HL), few intervention studies have evaluated the influence of HL on weight-related child outcomes. The prevalence of childhood obesity has increased significantly over the previous 4 decades, stabilizing over the past decade, with approximately one-third of US children being classified as obese (1). Obesity disparities persist between minority and low socioeconomic status children and their more advantaged counterparts (2). When considering children aged 8 to 12 years, parents and caregivers play an important role in providing eating and physical activity opportunities. Caregivers with low HL may be less likely to provide healthful eating and physical activity options for their children, making HL a potential contributing factor in childhood obesity (3).

HL can be defined as an individual’s capacity to obtain, process, and understand basic health information and services needed to make appropriate health decisions. Approximately 1 in 3 parents or caregivers have low HL (4). Furthermore, prevalence of low HL is even higher among low-income and racial/ethnic minority populations (5). Poor HL skills have consistently been linked with poorer health outcomes (6). In theory, intervention strategies that address HL may allow participants with low HL to process information at a similar rate to those with higher HL and thus benefit to the same or greater degree.

Most scientific literature on HL focuses on adult populations (6), although emerging literature demonstrates that the HL status of parents and caregivers can affect the health outcomes of children (4,6–8). Parents and caregivers play a crucial role in making informed decisions about diagnosis and treatment of health conditions, as well as establishing appropriate adherence and follow-up (4,6). Evidence also suggests that children of parents with high HL are more likely to have better health outcomes than children of parents with low HL (8). One systematic review found that adults with low HL are up to 4 times more likely than adults with high HL to exhibit negative health behaviors that affect child health (7). However, of the 23 included studies, most (n = 18; 78%) were cross-sectional, and none focused on child weight outcomes. Since this 2009 review, Sanders and colleagues have conducted intervention work addressing parental HL to prevent obesity among children younger than 24 months (3). However, no other known intervention studies have focused on parent or caregiver HL-sensitive strategies to improve child body mass index (BMI) *z* scores, including those among children aged 8 to 12 years, where the largest body of evidence-based childhood obesity treatment interventions exist.

Clearly void in the literature are initiatives to address child health inequities, including childhood obesity programs that incorporate design features and treatment strategies that meet the HL needs of parents (9). The use of HL universal precautions is one approach to address the HL needs of parents (10). The HL universal precautions is founded on the concept that, regardless of HL status, all individuals can benefit from simplified health information, reduced literacy demands of program components, reinforcement of key messages, and practice activities that promote skill development and self-management. There is a lack of research related to the use and effects of an HL universal precautions approach applied to childhood obesity treatment programs (6–8,10). Furthermore, there is insufficient evidence to conclude whether HL interventions can equitably reach and engage parents with low and high HL (11).

Guided by a community-based participatory research and systems-based approach, the Partnering for Obesity Planning and Sustainability Community Advisory Board (POPS-CAB) was initiated in 2012 to build community capacity to develop, implement, and sustain an evidence-based childhood obesity reduction initiative in the Dan River Region, a federally designated medically underserved area. In this health disparate region, the prevalence of childhood obesity is approximately 3 times higher than state and national averages. This initiative was funded by a planning grant mechanism from the National Institutes of Health, and the overall evaluation was guided by the RE-AIM (Reach, Effectiveness, Adoption, Implementation, Maintenance) framework. Preliminary primary outcomes analysis indicated that our intervention resulted in significant decreases in both child and parent weight status (12); however, the HL data were not examined. Therefore, this secondary data analysis explored whether parental HL influenced reach, attendance, and retention of and outcomes in a 3-month family-based childhood obesity treatment program in the Dan River Region. By applying an HL universal precautions approach in our research protocol and intervention strategies (10), we hypothesized that no significant differences would be found among children of parents with low versus high HL.

## Methods

This quasiexperimental trial, with pre–post program data, was tested in 3 waves from January 2013 through September 2015. The POPS-CAB included regional health care, public health, and parks and recreation partners, as well as academic partners. The POPS-CAB was involved in all research phases, including intervention selection and adaptation of the iChoose program, recruitment of families, program implementation, and data collection and evaluation (13).

Eligibility criteria for iChoose participation were English-speaking children aged 8 to 12 years with a BMI at or above the 85th percentile who resided in the Dan River Region. Exclusion criteria were children who had major cognitive impairments and contraindication for physical activity among children and parents. The primary recruitment methods included referral from the POPS-CAB health care partners who identified eligible children through medical record reviews, mailed study invitation letters, and called families to screen for basic demographic characteristics and interest in participating.

All study procedures were approved by the Virginia Tech institutional review board. Parents provided written informed consent, and children provided written assent. To compensate time to complete the data assessments, $25 gift cards were provided to children and parents at baseline and at the 3-month assessments.

iChoose was adapted from an evidence-based and family-based childhood obesity treatment program (14) to meet local needs and address feasibility for local community organization delivery. An HL universal precautions approach was applied when developing the research protocol and multicomponent program strategies (10,15–17). In this approach, it is assumed that all participants may have difficulty reading, comprehending, and acting on health information. An HL universal precautions approach is hypothesized to reduce the disparities in achieving significant outcomes between participants with low and high HL because of differences that often occur in traditional intervention approaches. The resulting 3-month multicomponent program and the applied a priori HL strategies are described in Table 1.

**Table 1:** iChoose Intervention Structure Overview and Application of Health Literacy Universal Precautions Strategies, Effect of Parent Health Literacy Status on Outcomes of a Family-Based Childhood Obesity Treatment Program, Virginia, 2013–2015

Components	Description of iChoose Components	Identified Health Literacy Universal Precaution Strategies	Application of Strategies to the iChoose Program
Small group family classes	Each of the 6 small group classes were approximately 2 hours in duration, with approximately 8 to 12 families per class. Classes included an interactive, didactic nutrition component and a physical activity component that engaged families in movement for about 20 minutes. For the behavioral component of each class, parents and children received separate training, with complementary, role- and age-specific objectives and activities.	1) Use visual and experiential learning techniques; 2) empower participants to ask questions by creating a shame-free environment; and 3) make an action plan.	Verbal presentations were accompanied by pictorial information presented in PowerPoint (Microsoft Inc) slides and handouts. Engaging hands-on activities and demonstrations were used to reinforce key messages. Group questions were encouraged and facilitated. At the conclusion of each family class, families set an exercise and nutrition goal for the next 2 weeks.
Workbooks	The parent workbook and child workbook were developed in 6 chapters to be used in conjunction with the small group classes. Each chapter was divided into components of nutrition, physical activity, and behavioral strategies and included the module objectives, educational content, a class activity, and homework.	1) Train community advisory board members to evaluate the quality of written materials; 2) assess readability and create written materials that are at a 5th-grade reading level or below; 3) assess understandability and acceptability of written materials; and 4) have participants provide feedback on written materials.	Community partners and researchers were trained on CDC’s Clear Communication Index (CCI) and the Suitability Assessment of Materials (SAM) to assess both understandability and cultural appropriateness of the materials (15,16). These assessments, along with focus group feedback from iChoose participants, resulted in workbook modifications, including simplified language and culturally appropriate language, images, and examples. The modified workbooks have a final readability rating of 90 out of 100 on the CCI, scored at a 5th-grade reading level on the Simple Measure of Gobbledygook, and achieved cultural relevance with 9.2 or 10 on the SAM.
Child newsletters	The 6 child newsletters were mailed the week following each small group family class.	1) Improve self-management by reminding participants of what they learned from each class; 2) encourage participants to stick to their action plans; and 3) provide support by linking participants to resources to avoid relapses (including each other as role models).	Child newsletters were designed to be action-oriented for behavioral recommendations, feature and highlight iChoose success stories, and provide fun educational activities to reinforce key behavioral messages from the previous class. Newsletters were personalized for each cohort with pictures from class of participants role-modeling healthy behaviors during classes. Newsletters also included special features for a current holiday and/or seasonal health tips to avoid relapses.
Physical activity sessions	Twenty-four physical activity classes (2 per week) were scheduled for 1 hour with the purpose of engaging the children in moderate to vigorous physical activity.	1) Demonstrate new activities and behaviors; and 2) empower participants to follow through on action plans.	Physical activity sessions focused on exposure to new activities and the opportunity to practice these activities. The objective was to build confidence and skill in meeting physical activity recommendations. Exercise during these sessions counted toward family action plans.
Telephone support calls	One week following each small group class, the parents received a telephone support call, delivered by a research or community staff member.	Use teach-back method to improve understanding of and adherence to behavioral recommendations discussed in the family classes and provided in workbooks.	The support calls incorporated teach-back and teach-to-goal strategies. To promote comprehension of learning objectives, parents were asked to explain, or teach back, key concepts from the prior small group class. When concepts were recalled incorrectly, the answers were provided and discussed with participants. Using a teach-to-goal approach, participants were given 2 additional opportunities to teach back the key concepts within the same call. This health literacy strategy was used to assess parent comprehension as well as to clarify and reinforce key messages.
Goal setting and self-monitoring	Goal setting occurred during the small group classes and was reinforced during the telephone support calls. Self-monitoring activities were incorporated in the small group and workbook activities.	1) Make action plans; and 2) follow up with participants to monitor progress on action plans and encourage self-monitoring.	Goal setting was used during the classes and calls to foster empowerment by setting new behavior change goals while recognizing barriers and potential solutions to barriers. Self-monitoring was used to promote self-management by increasing awareness of behaviors throughout the intervention.

Abbreviation: CDC, Centers for Disease Control and Prevention.

Program delivery responsibilities were shared among trained research and community staff, including one doctoral-level primary investigator, 4 trained graduate research assistants, one staff member from Parks and Recreation with a bachelor’s degree in exercise, and 4 registered nurses from the health department. Intervention fidelity was assessed and did not differ by research or community staff implementation.

### Measures

Following standardized techniques, trained research and community staff collected outcome data at baseline and 3 months postprogram. All questionnaires were administered by an interviewer. 

Parents reported sex, age, race, annual household income level, and education status during the initial screening. Attendance was tracked for all intervention components.

To assess reach, 3 validated HL screening items were used. These HL items asked participants to rate perceptions of their HL skills on a 5-point Likert scale. Items focused on the degree to which people need help in reading health care materials (18), can confidently complete medical forms (19), and perceive their reading ability (20). Responses were summed to produce a continuous score, ranging from 3 to 15, with higher scores indicating higher HL. For parents who enrolled in the study, the validated Newest Vital Sign (NVS) was used to explore the influence of HL on retention, attendance, and outcomes. This 6-item questionnaire, based on the Nutrition Facts Panel, can be on a continuous scale or collapsed to represent low (0–3 correct responses) or high (4–6 correct responses) HL categories (21).

A calibrated digital Tanita scale (model no. 310GS; Tanita Corporation of America, Inc) and research-grade SECA 213 portable stadiometer (Seca North America) were used to assess body weight and height, without shoes and in light clothing. BMI measurements and scores were calculated using established Centers for Disease Control and Prevention protocol.

Validated measures assessed self-reported behavioral outcomes among both children and parents. These measures were the Godin Leisure Time Exercise Questionnaire to assess physical activity behaviors (22); the Behavioral Risk Factor Surveillance System (BRFSS) 6-item brief screener to assess intake of fruit and vegetable servings; the Beverage Questionnaire to assess sugar-sweetened beverage consumption (23); and the BRFSS screen time questionnaire for children only. Quality of life was measured using the Pediatric Quality of Life Inventory for children (24) and the BRFSS Healthy Days module for parents.

### Data analysis

All data were entered into SPSS statistical analysis software (version 23.0; IBM Corporation), and validated scoring procedures were applied to compute outcome variable scores. Descriptive statistics were used to summarize demographic characteristics and attendance rates. Chi-square test of association or Fisher exact test and analysis of variance were used to compare reach, attendance, and retention rates between groups. Independent *t* tests were used to estimate differences in outcomes by parent HL status at baseline and at follow-up. Program outcome effectiveness analyses were conducted using Stata version 13.1 (StataCorp LP). Generalized linear mixed-effect methods were used to estimate both the main time effect and parental HL moderation effects on children’s and parents’ outcomes, which handled the cohort-nesting feature of our data. Because imputations can introduce severe biases when observation numbers are small, completers-only (ie, children and parents retained at 3 months) analyses are reported. Nonetheless, completers-only results yielded findings similar to those of last-observation-carried-forward simple intention-to-treat analyses. Significance was set at *P* < .05.

## Results

**Reach.** Health care partners mailed 586 mailed letters to parents of children, inviting them to participate; 29 children were determined to be ineligible. Of those eligible, 60% of parents were contacted and 40% were unable to be contacted after 6 call attempts over 2 weeks. Of 101 enrolled children, 52% were male, 60% were black, and 71% had Medicaid; mean age was 9.8 (standard deviation [SD], 1.3) years. Characteristics of eligible and enrolled children were not statistically different from the characteristics of the 456 eligible children who did not enroll. Seven families enrolled 2 eligible children.

Of 94 enrolled parents, most were female (93%) and black (60%); mean age was 39.7 (SD, 8.9) years. Most parents (49%) reported an annual household income of less than $25,000, and 39% had a high school education or less. NVS scores indicated that 32 (34%) parents had low HL and that 62 (66%) had high HL. Among eligible parents with HL screening data (n = 226), there was no significant difference in HL scores (*t*_224_ = −0.32, *P* = .75) between those who enrolled their child (n = 92; mean, 14.07; SD, 1.52) and those who did not enroll (n = 134; mean, 14.13; SD, 1.37). There were also no significant differences for race, education level, or annual household income among eligible parents who did and did not enroll children.

**Attendance.** Among the 101 enrolled children, the average attendance was 2.3 of 6 family classes, 5.6 of 24 physical activity sessions, and parental completion of 3.8 of 6 support calls. There were no significant differences in attendance rates by parental HL status.

Seventy-one (70%) children and 67 (71%) parents completed the 3-month assessment. There was no significant difference (*t*_92_ = 1.13, *P* = .26) in HL scores, as assessed with the NVS, between the baseline HL score of parents of children who were retained at the 3-month follow-up assessment (n = 67; mean, 4.10; SD, 1.80) and those who were lost to follow-up (n = 27; mean, 3.63; SD, 1.92).

**Effectiveness outcomes.** At baseline, screen time for video and computer games among children of parents with low HL was higher than for children of parents with high HL (*P* = .03) (Table 2). A trend for low reported minutes per week of moderate to vigorous physical activity was found among low HL parents (*P* = .08), but no significant differences were found in child or parent variables by parental HL status. At postprogram, there were no significant differences by parent HL status, and the differences between the significant and trending variables at baseline shrunk in magnitude (children screen time for video and computer games 1.1 to 0.6).

**Table 2:** Baseline and Postprogram Outcomes Among Children and Parents, Overall and by Parent Health Literacy Status (Completers Only)^a^, Effect of Parent Health Literacy Status on Outcomes of a Family-Based Childhood Obesity Treatment Program, Virginia, 2013–2015

Outcome	Overall			Parents With Low Health Literacy			Parents With High Health Literacy			Baseline Low vs High Health Literacy		Postprogram Low vs High Health Literacy	
	Baseline	Postprogram	Δ	Baseline	Postprogram	Δ	Baseline	Postprogram	Δ	Mean (SD) Difference	*P* b	Mean (SD) Difference	*P* b
	Mean (SD)												
**Children**													
Body mass index, *z* score	1.91 (0.45)	1.87 (0.49)	−0.04 (0.24)	1.88 (0.48)	1.82 (0.55)	−0.06 (0.24)	1.93 (0.44)	1.89 (0.47)	−0.05 (0.16)	−0.05	.65	−0.06	.62
Physical activity, minutes MVPA/wk	159.3 (167.2)	212.2 (173.4)	52.9 (224.5)	125.7 (164.5)	166.6 (172.7)	41.0 (217.3)	179.3 (167.7)	239.3 (170.4)	60.0 (231.3)	−43.4	.31	−63.8	.15
Fruit and vegetables, servings/d	3.2 (2.6)	3.0 (2.5)	−0.2 (3.0)	3.6 (3.2)	3.4 (2.9)	−0.2 (3.51)	3.0 (2.2)	2.8 (2.3)	−0.2 (2.7)	0.6	.38	0.5	.41
Sugar-sweetened beverages, oz/d	22.0 (22.0)	13.0 (14.7)	−9.1 (21.0)	20.4 (17.0)	12.6 (14.2)	−7.8 (20.5)	22.9 (24.2)	13.2 (15.1)	−9.7 (21.5)	−2.4	.67	−0.6	.81
Screen time, watch television^c^	3.1 (1.7)	2.8 (1.8)	−0.3 (1.7)	3.4 (2.1)	2.9 (2.0)	−0.5 (2.0)	2.9 (1.5)	2.7 (1.8)	−0.2 (1.6)	0.6	.25	0.2	.64
Screen time, play video game/computer^c^	2.2 (1.8)	2.2 (1.7)	−0.04 (1.8)	3.0 (2.1)	2.6 (1.9)	−0.4 (2.4)	1.9 (1.5)	2.0 (1.5)	0.1 (1.6)	1.1	.03	0.6	.18
Quality of life, total (100-point scale)	70.7 (14.8)	73.7 (12.3)	3.0 (10.8)	71.2 (12.8)	74.9 (9.4)	3.6 (12.2)	70.4 (15.8)	73.1 (13.5)	2.7 (10.2)	0.7	.85	1.7	.59
**Parents**													
Body mass index, kg/m^2^	36.39 (8.76)	36.12 (8.93)	−0.27 (1.00)	35.57 (7.45)	35.32 (7.44)	−0.25 (0.91)	36.79 (9.62)	36.50 (9.40)	−0.28 (1.05)	−1.21	.60	−1.18	.61
Physical activity, minutes MVPA/wk	86.0 (156.9)	196.4 (192.7)	110.4 (208.0)	50.3 (72.5)	221.1 (197.9)	170.8 (208.0)	100.9 (179.6)	186.0 (191.9)	85.1 (205.1)	−54.5	.08	37.6	.48
Fruit and vegetables, servings/d	2.5 (1.7)	3.1 (2.2)	0.6 (1.6)	2.3 (1.9)	3.3 (2.7)	1.0 (1.7)	2.6 (1.6)	2.9 (1.9)	0.4 (1.5)	−0.03	.55	0.4	.51
Sugar-sweetened beverages, oz/d	20.0 (22.9)	10.5 (10.0)	−7.0 (17.8)	23.6 (26.7)	14.2 (16.6)	−9.5 (19.9)	18.2 (20.8)	12.4 (14.0)	−5.8 (16.8)	5.9	.33	1.8	.65
Quality of life, no. unhealthy days in last 30 days	12.2 (11.2)	10.5 (10.11)	−1.6 (8.6)	12.2 (12.2)	10.2 (9.6)	−2.0 (9.0)	12.0 (10.7)	10.6 (10.4)	−1.4 (8.5)	0.5	.86	−0.7	.80

Abbreviations: MVPA, moderate to vigorous physical activity; SD, standard deviation.

a Seventy-one children completed the study (parental low health literacy, n = 23; parental high health literacy, n = 48); 67 parents completed the study (low health literacy, n = 22; high health literacy, n = 45). Health literacy items asked participants to rate perceptions of their health literacy skills on a 5-point Likert scale. Items focused on the degree to which people need help in reading health care materials (18), can confidently complete medical forms (19), and perceive their reading ability (20). Responses were summed to produce a continuous score, ranging from 3 to 15, with higher scores indicating higher health literacy. Sample sizes for each variable fluctuated slightly due to missing responses and outliers.

b *P* values calculated using independent *t* tests.

c Units: 0 = no screen time, 1 = <1 h/d, 2 = 1 h/d, 3 = 2 h/d, 4 = 3 h/d, 5 = 4 h/d, 6 = ≥5 h/d.

Significant main effects improvements were observed for child BMI *z* score, ounces of sugar-sweetened beverages, and quality of life (Table 3). Among main effect models for parents, significant improvements were observed for BMI, minutes of moderate to vigorous physical activity per week, servings per day of fruits and vegetables, and ounces of sugar-sweetened beverages per day. Parental HL status did not influence these outcomes, with the exception of child screen time for video game/computer (coefficient = 0.52; standard error [SE], 0.11; *P* < .001). Children of parents with low HL decreased their video game/computer screen time by 0.39 (2.35) units, whereas children of parents with high HL increased by 0.13 (1.61) units (1 unit equates to approximately 1 hour).

**Table 3:** iChoose Main Effects Among Children and Parents and Moderation Effects, by Parent Health Literacy Status, Effect of Parent Health Literacy Status on Outcomes of a Family-Based Childhood Obesity Treatment Program, Virginia, 2013–2015

Outcomes	Main Effect Coefficient (Robust Standard Error)^a^	*P* Value	Parent Health Literacy Moderation Effect Coefficient (Robust Standard Error)^a^	*P* Value
**Children**				
Body mass index, *z* score	−0.05 (0.02)	.01	0.01 (0.07)	.88
Physical activity, minutes MVPA/wk	52.88 (30.85)	.87	19.01 (69.65)	.78
Fruit and vegetables, servings/d	−0.16 (0.59)	.79	0.03 (0.81)	.97
Sugar-sweetened beverages, oz/d	−9.06 (3.17)	.004	−1.94 (4.71)	.68
Screen time, watch television^b^	−0.30 (0.18)	.08	0.32 (0.46)	.48
Screen time, play video game/computer^b^	−0.04 (0.24)	.86	0.52 (0.11)	<.001
Quality of life, total (100-point scale)	3.00 (2.74)	.009	−0.95 (4.83)	.84
**Parents**				
Body mass index, kg/m^2^	−0.28 (0.04)	<.001	−0.04 (0.29)	.89
Physical activity, minutes MVPA/wk	110.64 (40.74)	.007	−90.31 (56.42)	.11
Fruit and vegetables, servings/d	0.58 (0.27)	.03	−0.65 (0.41)	.12
Sugar-sweetened beverages, oz/d	−6.63 (0.37)	<.001	4.27 (5.21)	.41
Quality of life, no. unhealthy days in last 30 days	−1.93 (1.37)	.16	0.79 (1.10)	.47

Abbreviation: MVPA, moderate to vigorous physical activity.

a Generalized linear mixed-effect parametric models that control for cohort. Generalized linear mixed-effect parametric models that control for race and income do not substantially influence the main or moderation effect trends. These models are not presented because of missing income information and decreased sample size.

b Units: 0 = no screen time, 1 = <1 h/d, 2 = 1 h/d, 3 = 2 h/d, 4 = 3 h/d, 5 = 4 h/d, 6 = ≥5 h/d.

## Discussion

In our universal HL precautions approach, we developed research protocol and intervention materials for the multicomponent iChoose program to reduce the literacy demands and address the HL needs of the targeted families. Therefore, it was important to discover that HL status had no influence on reach, attendance, and retention and little influence on postprogram outcomes (with the exception of child video game/computer screen time). Although few significant gaps existed between parents with low and high HL at baseline, our approach provides preliminary evidence that inequalities were not generated as a result of iChoose. These exploratory findings address notable gaps in the literature (4,6–8) and in a health disparate region with high childhood obesity prevalence.

Our intervention significantly decreased child weight status within the range of effects documented in previous efficacy trials (9). However, prior trials did not examine differences by parent HL status, leaving little opportunity for direct comparisons. Most information related to relationships between parental HL status and child outcomes is limited to cross-sectional studies (6–8,25,26), with few in the context of childhood obesity (27,28). For example, odds of obesity among children decrease with higher parent HL status (27), and parent nutrition literacy is associated with higher child Healthy Eating Index scores (28). Beyond these 2 studies, no other known studies have examined the relationship between parent HL status and child weight or obesity-related behaviors such as sugar-sweetened beverage consumption, fruit and vegetable consumption, screen time, and physical inactivity. When available, results from the ongoing intervention research by Sanders and colleagues to address parental HL to prevent obesity among children younger than 24 months will advance understanding of the role of parental HL on child obesity–related outcomes (3).

The fact that there was no main intervention effect on child video game/computer screen time but that screen time was influenced by parent HL status exemplifies the importance of our analysis. Screen time was addressed in week 6 group family sessions and week 7 and 8 support calls, and families were encouraged to develop a calendar to limit screen time to less than 2 hours per day. At baseline, video game/computer screen time among children of parents with low HL was approximately 1 hour per day higher than among children of parents with high HL. Children of parents with low HL decreased their time by approximately 24 minutes per day while children of parents with high HL increased by approximately 6 to 9 minutes per day, resulting in a nonsignificant difference of about one-half an hour at the 3-month follow-up. This finding shows that the differential gap in video game/computer screen time between children of parents with low versus high HL at baseline was narrowed at the follow-up. Although we interpret these results cautiously because this is a pilot study, our findings provide the foundation for potentially fruitful research studies to identify and address child health disparities related to parent HL status.

Beyond parent or caregiver HL interventions, the broader literature indicates notable advancements in experimental HL approaches (5). However, many research gaps persist in experimental HL research that targets disease self-management and health promotion (11). First, few experimental or quasiexperimental studies report on how participant HL status influences reach, adherence, or retention (11). Second, few experimental or quasiexperimental studies have explored how HL status influences weight outcomes in behavioral interventions, and, among those, findings have been mixed. Our study attempts to address these documented areas of opportunities for research and practice. Ideally, future family-based childhood obesity treatment programs should seek to improve outcomes for all families and narrow any existing gaps in outcomes between families of low and high HL (6).

Interpretations of this exploratory and secondary data analysis are limited by the quasiexperimental design, small sample size, and short follow-up period. Also, although validated measures of HL were used, these measures may not capture the full complexity of HL skills. Despite these limitations, this research, conducted as part of a planning grant initiative, provides preliminary data to advance the next phases of childhood obesity treatment research in the targeted underserved region and can inform the work of other practitioners and researchers. Evidenced by a seminal review of HL studies, pilot testing intervention strategies before implementing them on a full scale leads to effective interventions (5). As one example, although parent HL status did not influence participation, overall participation was lower than desired. Lessons learned from this planning grant, including summative exit interviews of parents, will be used to further develop culturally relevant engagement and retention strategies.

Future studies should include a more robust randomized controlled study design, longer follow-up, and an adequate sample powered to detect HL moderation effects and to control for other potentially relevant demographic factors. There is also opportunity to test which HL strategy features, either alone or in combination, are most successful in mitigating the effects of low HL on targeted outcomes (5). Finally, exploring the influence of child HL and child obesity outcomes should be a priority. Literature is beginning to emerge in this area (29). However, there are issues and opportunities related to use of validated HL measures in children, especially younger children, such as the 8- to 12-year-olds included in this study (30).

This study provides preliminary evidence on the utility of applying an HL universal precautions approach in a family-based childhood obesity treatment program. By incorporating design features and obesity treatment strategies that address the HL needs of all parents in our study, children of parents with low HL participated and benefited from the intervention similarly to children of parents with high HL. Although future studies are needed to further explore the generalizability of our findings, public health specialists are encouraged to use a universal HL precautions approach to meet the needs of parents and caregivers, especially in underserved areas where resources, health information, and behavioral skills training opportunities are limited (4,6–8).
